# Anti-inflammatory effects of *Polygonum minus (Huds)* extract (Lineminus™) in in-vitro enzyme assays and carrageenan induced paw edema

**DOI:** 10.1186/1472-6882-14-355

**Published:** 2014-09-25

**Authors:** Annie George, Sasikala Chinnappan, Meena Chintamaneni, Vandana Kotak C, Yogendra Choudhary, Thomas Kueper, Ammu K Radhakrishnan

**Affiliations:** Biotropics Malaysia Berhad, Lot 21 Jalan U1/19, Section U1, Hicom-Glenmarie Industrial Park, 40150 Shah Alam, Selangor Malaysia; Division of Toxicology and Clinical Affairs, Ethix Pharma, Karbala Road, Bilaspur, 495001 Chhattisgarh India; Stephanstrasse 28, 48734 Reken, Germany; Pathology Division, Faculty of Medicine and Health, International Medical University, Kuala Lumpur, Malaysia

**Keywords:** Inflammation, *Polygonum minus* (Huds), Paw edema, Cyclooxygenase, Lipooxygenase, Secretory phospholipase-A2

## Abstract

**Background:**

The study was aimed to evaluate the anti-inflammatory activity of ethanolic and aqueous extracts of *Polygonum minus* (Huds) using in vitro and in vivo approaches.

**Methods:**

The in vitro tests used to evaluate ethanolic extract are cyclooxygenase-1 (COX-1), cyclooxygenase-2 (COX-2), lipooxygenase (5-LOX), secretory phospholipase-A2 (sPLA2) inhibition assay whilst the in-vivo effect was measured by the ability of aqueous extracts to reduce paw edema induced by λ-carrageenan, in rats.

**Results:**

The ethanolic extract inhibited the activities of 5-LOX and COX-1(p < 0.05) whilst the inhibitory effect on COX-2 was only moderate. A marked inhibition of 5-LOX was observed at 30 μg/ ml. The extract did not inhibit the activity of sPLA2. The ability of the ethanolic extracts of *Polygonum minus* to inhibit both 5-LOX and COX, prompted a study to evaluate the effects of using an aqueous extract of *Polygonum minus***(**Lineminus^TM^); as this would be more suitable for future clinical testing. The anti-inhibitory activity of the aqueous extract from this plant was evaluated using a rat model where inflammation was induced in the paws by injection of λ-carrageenan. The aqueous extracts from *Polygonum minus* administered at doses of 100 and 300 mg/kg body weight (b.w.), significantly (p < 0.01) reduced paw edema induced by λ-carrageenan in the experimental model, at 4 h compared to the vehicle control. Furthermore, administration of 100 mg/kg b.w. or 300 mg/kg b.w. completely reduced inflammation of the paw 4 h after injection.

**Conclusion:**

These findings suggest that aqueous extract of *Polygonum minus* possesses potent anti-inflammatory activities.

## Background

Inflammatory reaction, typically characterized by redness, swelling, heat, and pain is one of the most important host defense mechanisms against invading pathogens. However, persistent or over-inflammation can result in tissue damage and possibly failure of vital organs. In a number of pathological conditions, acute and/or chronic inflammation can lead to acute or chronic inflammatory diseases such as septic shock, rheumatoid arthritis, gastritis, and atherosclerosis [1, 2]. Over a period of time, inflammation due to trauma, genetic predisposition, stress and age can cause damage to cells of the body, thus releasing various membrane components that can activate the inflammatory process. The phospholipids liberated from the lipid bilayer membrane can be converted to arachidonic acid (AA) by the enzyme phospholipase A2 (PLA2). Arachidonic acid produced plays an important role in many metabolic pathways and is useful when produced in moderation. However in cases of severe inflammation such as joint damage, the AA is produced in excess [3]. Excess AA is converted by the cyclooxygenase (COX) and lipooxygenase (LOX) pathways into powerful inflammatory substances such as prostaglandins (PG) and leukotrienes (LT), respectively [4, 5].

In the past few years, there has been concerted research focusing on the LOX pathway as controlling this pathway can play an important role in the relief of joint pain. The LOX pathway is a parallel inflammatory pathway to the COX pathway, where the AA produced is converted to LT, one of the strongest chemotactic agent produced in the body [6, 7]. If left unregulated, these inflammatory pathways can cause joint damage. Cyclooxygenase-1(COX-1) and 5-lipooxygenase (5-LOX) are key enzymes involved in the formation of pro-inflammatory mediators such as eicosanoids from AA [8]. The COX-1 is constitutively expressed in many tissues and PGs produced by the action of COX-1 mediate housekeeping functions such as cytoprotection of gastric mucosa, regulation of renal blood flow and platelet aggregation, mostly through formation of eicosanoids from AA [9]. The 5-LOX enzyme plays a key role in the metabolism of AA to produce leukotrienes. Several studies suggest that there is a link between 5-LOX and carcinogenesis due to inflammation in human and animals [10]. Leukotrienes also play crucial roles as mediators in allergy and inflammation [11]. In addition, these pro-inflammatory mediators are also linked to some of the pathophysiological conditions of the brain such as cerebral ischemia, brain edema and brain tumors due to increased permeability of the blood–brain barrier (BBB) [12]. The 5-LOX enzyme is expressed in human brain tumors [10], hence it may play a role in inducing brain edema which causes brain tumor.

In the recent years, the use of herbal remedies for the treatment of inflammatory disease has been gaining momentum [13]. There has been some concern over the use of COX-2 inhibitors ie rofecoxib and valdecoxib for therapeutic interventions, causing some of these therapeutic products to be either withdrawn or made to carry a warning by the Food and Drug Authority (FDA) of the USA [14, 15]. Due to risk of cardiovascular and skin related toxicities, rofecoxib and valdecoxib were withdrawn from the market in September 2004 and March 2005 respectively [16]. On the other hand, inhibitors of the 5-LOX enzyme that are of herbal origin are reported to offer significant relief and do not appear to have any adverse effects. Therefore, 5-LOX inhibitors of plant origin are gradually becoming the preferred choice of treatment for some of the diseases due to chronic inflammation [17, 18].

*Polygonum minus* [(Huds) (Polygonaceae)] is a small herbaceous plant commonly known in Malaysia as Kesum [19]. This plant is a small, annual, slender, glabrous and erect to ascending herb, with tall or long branches [19]. This plant has a sweet and pleasant aroma and is commonly used by Malaysians as a flavoring ingredient [20] in Malaysian local dishes such as Laksa, Nasi Kerabu and Nasi Ulam [21]. Traditionally, this plant has been used to treat digestive disorders and dandruff. It is also used in the perfume industry because of its volatile oil [22, 23]. It has proven to be a potent natural source of antioxidants and there are several reports that claim that it has a high level of free radical scavenging activity and reducing power [24]. Flavonoids have been reported in *Polygonum minus* [25] and these phenolic compounds have been associated with anti-inflammatory effects [26], but none reports anti-inflammatory effects of *Polygonum minus*.

Meanwhile, safety studies such as acute and sub-acute toxicity of aqueous extract of *Polygonum minus* (biotropics®PM101), in Wistar rats has shown that the no-observed-adverse-effect-level (NOAEL) following oral administration for 28 days, to be more than 1000 mg/kg body weight [27]. The aqueous, ether and ethanol extracts of *Polygonum minus* have also been extensively studied for their phenolic content, anti-oxidant and cytoprotective activity [28], hence this study is to investigate whether *Polygonum minus* extracts possess anti-inflammatory activity as a result of flavonoids content and antioxidant property.

In the present study, we investigated the anti-inflammatory activities of extracts obtained from the aerial parts consisting stem and leaves of *Polygonum minus* using in vitro and in vivo approaches.

## Methods

### Chemicals and drugs

The chemicals λ-carrageenan, diclofenac sodium and carboxy methyl cellulose (CMC) were purchased from Sigma-Aldrich Chemical Co. in Mumbai, India. Cyclooxygenase (COX) Inhibitory Screening Assay Kit, Lipoxygenase (5-LOX) Inhibitor Screening Assay Kit, Secretory Phospholipase A2 (sPLA2) (Type V) Inhibitor Screening Assay Kit, Diclofenac, Nordihydroguaiaretic acid (NGDA) and Arachidonic Thioester Phosphatidylcholine (TEPC) were procured from Cayman Chemical Company (Tallinn, Estonia).

### Plant material

*Polygonum minus* was procured from Biotropics Malaysia Berhad, Malaysia. The plant material was identified on the basis of exomorphic characters and review of literature by a Taxonomist from Institute Bio Science, University Putra Malaysia (UPM). The voucher specimen of the plant (SK 2077/12) was deposited in the Herbarium, Institute Bioscience UPM of Malaysia. The aerial parts comprising the stem and leaves were extracted using different extraction techniques to produce ethanol extract for in-vitro assays and aqueous extract for in-vivo assay.

### Extract preparation for in-vitro assays

The aerial parts were dried by oven drying at a temperature of 40°C for 48 hours and shredded to 2 to 5 cm in size. Then, 100 g of the milled plant material were subjected to an organic extraction with 750 ml 90%:10% (v/v) ethanol: water mixture with aid of sonication at maximum temperature of 40°C for 30 minutes. The solvents were reduced by evaporation on a rotary evaporator at reduced pressure (15 mbar) to approximately 50 ml. The remaining mixture was added with water to a final volume of 100 ml. The mixture was extracted two times in liquid/liquid separation with 150 ml heptane to remove the lipophilic fraction, followed by three fold extraction in liquid/liquid separation with 150 ml ethyl-acetate to obtain polar and semi-polar fractions. The ethyl acetate phase was evaporated to dryness. After which, 50 mg of ethylacetate extract was dissolved in 100 μl methanol and applied on SPE column (RP-18), which was equilibrated with water and further eluted with 3 ml of 20%, 40% and 70% acetonitrile. The fractions that were eluted with 20%, 40% and 70% acetonitrile were collected, combined and evaporated to dryness.

### Extract preparation for in-vivo anti-inflammatory activity

Aqueous extract was prepared for in vivo testing to mimic as close as possible the human consumption of the stems and leaves in cooking and traditional application. Hence, the aerial parts (comprising stem and leaves) were dried by oven drying at a temperature of 40°C for 48 hours and shredded to 2 to 5 cm in size. A total of 100 g dried aerial parts were then subjected to percolation using 1000 ml of purified water and extracted at a temperature of about 80°C. The extract was further filtered, concentrated using rotary evaporator with the water bath temperature at 65°C and freeze-dried. The extract was a standardised propriety extract with quercetin-3-glucuronide (0.59%) and quercitrin (0.27%) as marker compounds and trademarked as Lineminus™ [29]. The dried crude extract was dissolved in 0.5% CMC (carboxymethyl cellulose) solution to produce two test doses of 100 mg/kg and 300 mg/kg respectively prior to pharmacological testing.

### Enzyme assays COX-1 & COX-2, 5-LOX and sPLA2

The enzyme assays are part of a preliminary screening program for anti-inflammatory activities to justify further in vivo test in the area of inflammation. Hence the ability of *Polygonum minus* extract to inhibit COX-1 & COX-2, 5-LOX and sPLA2 were evaluated.

### Cyclooxygenase (COX-1 and COX-2) inhibition assay

The extract was dissolved in 100% DMSO to prepare a stock concentration of 10 mg/ml. The extract was tested in triplicates at 30 and 100 μg/ml using a commercial COX inhibitory screening assay kit as recommended by the manufacturer (Cayman test kit-560131, Cayman Chemical Company). The COX inhibitor screening assay directly measures the amount of Prostaglandin2α produced in the cyclooxygenase reaction. Diclofenac (MW = 296.14) was run as the positive control for inhibition of COX-1 and COX-2. A volume of 10 μl each of test extract and vehicle were diluted to 20 μl with 0.1 M Tris–HCl pH 8.0 and pre-incubated with the enzyme at 37°C for 15 minutes prior to the addition of AA. The reaction is initiated by addition of 10 μl 10 mM AA and the tube was incubated at 37°C for another 2 minutes. Reaction was terminated by addition of 50 μl 1 N HCl and saturated stannous chloride. Assays were performed using 100 units of ovine COX-1 and human recombinant COX-2. An aliquot is removed and the prostanoid produced is quantified spectrophotometrically via enzyme immunoassay (EIA).

### Lipooxygenase (5-LOX) inhibition assay

The extract was tested in triplicates at 30 and 100 μg/ml using the LOX inhibitor screening assay kit using the protocol recommended by the manufacturer (Cayman test kit-766700, Cayman Chemical Company). This assay measures the hydroperoxides generated from incubating a 5-LOX enzyme with its substrate, AA. Nordihydroguaiaretic acid (MW = 302.36) was used as the positive control. A volume of 10 μl each of test extracts and vehicle were pre-incubated with 90 μl 5-LOX enzyme in a 96-well plate. The reaction was initiated by addition of 10 μl 1 mM AA and the plate was shaken for 5 minutes. Then, 100 μl of chromogen from the test kit was added to stop enzymic reaction and for color development. The plate was placed on a shaker for another five minutes and absorbance at 490 nm was measured using microplate reader.

### Secretory Phospholipase A_2_ (sPLA2) inhibition assay

The extract was tested in triplicates at 30 and 100 μg/ml using the sPLA2 (Type V) inhibitor screening assay kit as recommended by the manufacturer (Cayman test kit-10004883, Cayman Chemical Company). This assay kit contains a human recombinant Type V sPLA2 and reaction mixtures. Arachidonic thioester phosphatidylcholine was used as the positive control. A total of 10 μl each of test extract and vehicle were pre-incubated with 25 mM Tris–HCl buffer pH 7.5 containing 10 μl enzyme in a 96-well plate. The reaction was initiated by addition of 200 μl 1.66 mM Diheptanoylthio-PC and the plate was shaken for 30 seconds and incubated at 25°C for 15 minutes. Further, 10 μl 5,5′-dithio-bis-(2- nitrobenzoic acid) (DTNB) was added to stop the enzymatic reaction and to allow for color development. The plate was placed on a shaker for one minute to mix and absorbance was measured at 405 nm using plate reader.

### Experimental animals

Thirty two male and female Wistar albino (WA) rats aged between 8–10 weeks and weighing between 200–245 g were allowed to acclimatize into the standard laboratory conditions. The rats were placed in a room with controlled temperature (22 ± 1°C), relative humidity (54 – 68%) and with 12 h light/12 h dark cycles for one week before they were used in the study. Animals were provided with Nutrilab rodent diet (M/s Provimi Animal Nutrition Pvt. Ltd. India) and aquaguard water ad libitum. The experimental protocol was approved by the Institutional Animal Ethics Committee (IAEC) of SPTM, India [vide approval No. BIO/IAEC/479, dated September 03, 2012]. The study was conducted in accordance with the recommendation of the IAEC as well as the “Purpose of Control and Supervision of Experiments on Animals (CPCSEA)” guidelines for laboratory animal facility published in the Gazette of India, December 15^th^ 1998.

### Carrageenan-induced paw edema animal model

The test was conducted according to method based on previous published study [30]. Briefly, 0.1 ml carrageenan was injected into the sub-plantar region of the left hind-paw of each rat. The volume (ml) of induced edema was measured using a digital plethysmometer (IITC, USA). The rat’s foot pad became edematous soon after the injection of carrageenan. The rats were then randomly divided into four groups of eight (n = 8) rats each. The rats in the four experimental groups were fed with the various test agents 30 min after the carrageenan injection; (i) vehicle control (CMC 0.5%); (ii) positive control [diclofenac sodium (10 mg/kg)]; (iii) *Polygonum minus* aqueous extract (100 mg/kg) and (iv) *Polygonum minus* aqueous extract (300 mg/kg); respectively. The paw volume of each rat was measured at different time intervals (0, 2, 4, and 6 h) after carrageenan injection using this instrument. The percentage inhibition was calculated using the formula 100 (1-Vt/Vc), where Vc was the mean edema volume observed in the control group whilst Vt was the edema volume measured in the test groups [31]. All data of each group were expressed as mean ± SEM (n = 8). Statistical analyses were performed with ‘Graph pad prism v4’ software and were carried out using one way ANOVA followed by Dunnet post-hoc test. Statistical significance was set at p ≤ 0.05.

## Results

### Effect of Polygonum minus extract on in-vitro anti-inflammatory assays

*Polygonum minus* ethanolic extract showed dose-dependent inhibition of COX-1 and COX-2 with 100 and 25% inhibition respectively at 100 μg/ml (Table 1). Inhibition of 5-LOX at both tested doses (30 and 100 μg/ml) was 100%. The ethanolic extract from *Polygonum minus* did not show any inhibitory activity on the sPLA2 enzyme at both the concentrations (30 and 100 μg/ml) tested. The ethanolic extract from *Polygonum minus* inhibited COX-1, COX-2 and 5-LOX enzymatic activities.

**Table 1:** **Effect of** ***Polygonum minus*** **extract on COX-1 & COX-2, 5-LOX and sPLA2 inhibition**

***Receptor/enzyme***	***Source***	***Substrate***	***% Inhibition of standard***		***% Inhibition of Polygonum minus at doses***	
			***Standard***	***% of inhibition***	***30*** μ***g/ml***	***100*** μ***g/ml***
COX-1	Ovine platelets	Arachidonic acid	Diclofenac (1 μM)	100	30	100
COX-2	Human recombinant	Arachidonic acid	Diclofenac (1 μM)	100	00	25
5-LOX	Human recombinant Lipooxygenase	Arachidonic acid	NGDA (100 μM)	100	100	100
sPLA2	Human recombinant Type V sPLA2	Diheptanoyl thio-PC	TEPC (20 μM)	90	00	00

### Carrageenan-induced paw edema animal model

Injection of λ-carrageenan into the sub-plantar region of the left hind-paw rapidly induced paw edema. A significant (p < 0.01) reduction of paw edema volume was observed in the rats that were fed with the aqueous extract from *Polygonum minus* (100 and 300 mg/kg b.w.) after 4 h compared to the rats that were fed with the vehicle control (Table 2). These findings indicate that the aqueous extract of *Polygonum minus* posses potent anti-inflammatory properties. These findings show that the aqueous extracts (100 and 300 mg/kg b.w.) of *Polygonum minus* inhibited the inflammatory processes induced by the injection of λ-carrageenan in the paw of rats after 4 h of oral administration.

**Table 2:** **Effect of** ***Polygonum minus*** **extract on paw volume and percentage inhibition in carrageenan induced paw edema**

***Groups & Dose***	***Mean paw volume ± SEM (mL) (% Inhibition*** ^***a***^ ***)***			
	***0 hr***	***2 hr***	***4 hr***	***6 hr***
Vehicle control (0.5% CMC) 0 mg/kg b.w.	1.35 ± 0.03	1.46 ± 0.03	1.50 ± 0.03	1.42 ± 0.04
Standard (diclofenac)	1.38 ± 0.04	1.50 ± 0.02	1.37 ± 0.03^*^	1.41 ± 0.04
10 mg/kg b.w.	(-2.22%)	(-2.74%)	(8.66%)	(0.70%)
*Polygonum minus* aqueous extract	1.33 ± 0.02	1.47 ± 0.04	1.32 ± 0.03^**^	1.40 ± 0.05
100 mg/kg b.w.	(1.48%)	(-0.68%)	(12.00%)	(1.41%)
*Polygonum minus* aqueous extract	1.38 ± 0.03	1.42 ± 0.02	1.34 ± 0.02^**^	1.35 ± 0.04
300 mg/kg b.w.	(-2.22%)	(2.74%)	(10.67%)	(4.93%)

Values are expressed as mean ± SEM, n = 8 (*4Male + 4Female*); (-) = No inhibition.

^a^Inhibition is reported as a percentage compared to control.

*Vehicle control vs standard (p < 0.05).

**Vehicle control vs *Polygonum minus* (100 mg/kg) / *Polygonum minus* (300 mg/kg) (p < 0.01).

## Discussion

Dual 5-LOX/COX inhibitors are potential new drugs to treat inflammation. These agents act by blocking the formation of both PG and LT but appear to have no measurable effects on the formation of lipoxin. These types of inhibitors of inflammation with dual functions help to avoid some of the disadvantages of selective COX-2 inhibitors [32].

Leukeotrienes (LTs) also play a major part in the inflammatory process [33]. These compounds are synthesized via the lipooxygenase pathway with the help of the 5-LOX enzyme. The present study shows that *Polygonum minus* extract has anti-inflammatory effects that can inhibit both the COX-2 and 5-LOX enzymes. The *Polygonum minus* has been reported to be rich with flavonoids [34, 35]. Flavonoids can interrupt the oxidative generation of AA from phospholipids and reduce the downstream production of inflammatory metabolites from AA metabolism, oxidative damage, and induction of inducible inflammatory pathways due to their potent antioxidant capacity [36]. Based on a published paper [37] it was found that a wide variety of flavonoids modulate the activities of AA metabolizing enzymes such as PLA2, COX, and 5-LOX. The *Polygonum minus* aqueous extract used in this study was reported to possess high antioxidant content demonstrated by the ORAC score of between 16,000 to 35,000 μmol TE/g [29, 38]. Other studies showed that flavonoids with antioxidant capacity could reduce the cellular conversion of AA to MDA (malondialdehyde) in patients suffering from chronic inflammation [39]. Hence, it is possible that the high antioxidant capacity and flavonoids content of the extract used, have contributed to the anti-inflammatory effects seen in in vitro testing.

In the present study the expression of reactive oxygen species (ROS), TNF-α and nuclear factor (NF)-κBprotein were not evaluated but could be expected by the well-established antioxidant property of *Polygonum minus*, the current study that evaluated its anti-inflammatory activity and when comparing to the closely related *Polygonum hydropiper,* also known as laksa leaf in Singapore. Anti-inflammatory effects in vitro and in vivo for *Polygonum hydropiper* were reported, demonstrated by the inhibition of mRNA expression of pro-inflammatory genes such as COX-2, TNF-α, nuclear factor (NF)-κB and PG [40]. A parallel to this relative of *Polygonum minus* was drawn due to molecular systematic studies of the two showing 100% similarity using molecular genetic marker derived from four accessions of *Polygonum minus* [41]. These results suggest that *Polygonum minus* extract may have also inhibited formation of LTs from 5-LOX and may prevent accumulation of these key inflammatory factors, which contribute to tissue damage through a putative 5-LOX shunt seen with NSAIDs. The current experiments demonstrate that, unlike NSAIDs, *Polygonum minus* extract does not only inhibit COX metabolism of AA to PGs rather it acts via inhibition of both COX and 5-LOX enzyme activity. Other well researched flavonoids, such as green tea catechins and quercitin have been shown to inhibit sPLA2 thus modulating the generation of AA from membrane phospholipids [42, 43]. The *Polygonum minus* extract in the tested doses did not inhibit PLA2 activity in this study. The result suggests that *Polygonum minus* extract does not have the ability to modulate the generation of AA from membrane phospholipids produced by the destruction of tissue. Instead, it appears that *Polygonum minus* extract might possess potent active principles, which inhibits 5-LOX enzyme. With this point of view, the anti-inflammatory activity of *Polygonum minus* extract was evaluated using an established rat model to study inflammation. This rat model is widely used to screen the ability of new anti-inflammatory agents to reduce local edema induced in the rat paw by injection of an irritant agent [44]. Carrageenan induced edema has been commonly used as an experimental animal model for acute inflammation and is believed to be biphasic. The early phase (1–2 h) of the carrageenan model is mainly mediated by histamine, serotonin [45]. Flavonoids including quercetin as identified in the aqueous extract used in this study, have been reported to possess inhibitory effect on histamine release in mast cells which is an anti-inflammatory reaction [46]. The second phase of the carrageenan model is related to the release of prostaglandins and bradykinins [45, 47]. The *Polygonum minus* aqueous extract showed significant anti-inflammatory effect in λ-carrageenan-induced rat paw edema around 4 hours after administration of carrageenan. The maximum inhibition takes place around 4 hours during which these anti-inflammatory mediators are expected to be released. An anti-inflammatory effect was also observed in vivo in another closely related plant species, *Polygonum hydropiper*, which is also known as Kesum in Malay [40].

## Conclusion

These studies provide evidence to show that the *Polygonum minus* exert an anti-inflammatory effect by inhibiting both COX and 5-LOX activity, which was studied using an in-vitro model and further qualified in vivo.

## Abbreviations

COX-1: Cyclooxygenase-1
COX-2: Cyclooxygenase-2
5-LOX: lipooxygenase
sPLA2: Secretory phospholipase-A2
B.W.: Body weight
AA: Arachidonic acid
PG: Prostaglandins
LT: Leukotrienes
BBB: Blood–brain barrier
FDA: Food and Drug Authority
CMC: Carboxy methyl cellulose
NGDA: Nordihydroguaiaretic acid
TEPC: Thioester Phosphatidylcholine
UPM: University Putra Malaysia
EIA: Enzyme immunoassay
DMSO: Dimethyl sulfoxide
DTNB: 5,5′-dithio-bis-(2 nitrobenzoic acid)
WA: Wistar albino
IAEC: Institutional Animal Ethics Committee
CPCSEA: Committee for the Purpose of Control and Supervision of Experiments on Animals
PGH2: prostaglandin H2
FLAP: 5-LOX activating-protein
ROS: Reactive oxygen species
MDA: malondialdehyde
PLA2: Phospholipase A2 activity.
